# BmHR3 Is Essential for Silk Gland Development and Silk Protein Synthesis in Silkworms (*Bombyx mori*)

**DOI:** 10.3390/insects16040369

**Published:** 2025-04-01

**Authors:** Yao Ou, Qin Luo, Wenhui Zeng, Yiyun Tang, Jie Hu, Chun Liu, Yan Ma, Hanfu Xu

**Affiliations:** 1 State Key Laboratory of Resource Insects, College of Sericulture, Textile and Biomass Sciences, Southwest University, Chongqing 400715, China; oy101118@163.com (Y.O.); luoqin0325@163.com (Q.L.); wenhui202503@163.com (W.Z.); 13164440186@163.com (Y.T.); hujie980920@163.com (J.H.); mlliuchun@163.com (C.L.); 2 Chongqing Sericulture Science and Technology Research Institute, Chongqing 400715, China

**Keywords:** ecdysteroid signaling, fibroin promoter, genetic manipulation, nuclear receptor, tissue-specific regulation

## Abstract

The steroid hormone 20-hydroxyecdysone (20E), which is known to regulate insect molting and metamorphosis, is crucial for the normal development of silk glands (SGs) in the silkworm *Bombyx mori*. However, how the 20E signaling pathway and its core members function in the SG remains largely unclear. Here, we report that the orphan nuclear receptor BmHR3, a 20E-response factor, plays an essential role in regulating SG development and silk protein synthesis. First, we showed that tissue-specific *BmHR3* overexpression and knockout result in severe developmental defects in posterior silk glands (PSGs). Second, we revealed that *BmHR3* dysfunction in PSGs dramatically represses the transcription of silk fibroin protein-coding genes, thereby inhibiting fibroin protein synthesis. Finally, we confirmed that BmHR3 can regulate fibroin protein-coding gene expression via direct and indirect mechanisms. This study elucidates the vital function of BmHR3 in *B. mori* SG and provides valuable information for thoroughly understanding the regulatory roles of 20E signaling in specialized insect organs.

## Simple Summary

*Bombyx mori* is one of the most important insects, and it is characterized by its remarkable ability to produce natural silk proteins. Consequently, identifying key factors that regulate *B. mori* silk-secreting organs and silk protein synthesis is very important. In this study, the function of the 20E-response factor BmHR3 in the *B. mori* silk gland was analyzed. Tissue-specific *BmHR3* overexpression and knockout dramatically impaired silk gland development and silk protein synthesis. BmHR3 regulates the expression of silk fibroin protein-coding genes by directly binding to their promoters and by indirect mechanisms. These results reveal the indispensability of BmHR3 in the *B. mori* silk gland, highlighting the regulatory roles of 20E-response factors in insect silk-secreting organs.

## 1. Introduction

The silkworm *Bombyx mori* (Lepidoptera: Bombycidae) is a domesticated lepidopteran insect renowned for its ability to produce silk, a natural protein resource with broad applications in biomedicine and materials science [1,2,3]. The silk glands (SGs) of silkworms are unique organs that synthesize and secrete silk proteins. SGs are divided into three functional regions: the anterior silk gland (ASG) for filament transport, the middle silk gland (MSG) for sericin synthesis, and the posterior silk gland (PSG) for fibroin synthesis [4,5]. Silk production is tightly regulated by hormonal signals, particularly 20-hydroxyecdysone (20E) and juvenile hormones (JHs) [6,7,8]. While JHs modulate larval growth and silk gland maintenance [8,9,10], 20E signaling—via its receptor complex (EcR/USP)—orchestrates metamorphosis and tissue remodeling by activating downstream transcription factors [11,12,13,14]. However, the roles of specific 20E-response factors in SG development remain poorly characterized.

In *B. mori*, key 20E-response factors such as BmFtz-f1 and E74A regulate silk synthesis through direct promoter binding and JH antagonism. For example, BmFtz-f1 regulates *fibH* transcription by binding to its promoter and counteracts the effect of Bmdimm on *fibH* [15,16], while E74A relieves the repression of JH signaling on FMBP-1, a fibroin modulator [17,18,19]. These findings underscore the importance of 20E signaling in silk production, yet the involvement of other nuclear receptors, particularly HR3—a conserved 20E-response factor—remains unexplored.

The orphan nuclear receptor HR3, an ortholog of mammalian retinoic acid-related orphan receptors (RORs), is a key mediator of 20E signaling. In *Drosophila melanogaster*, HR3 coordinates metamorphosis by regulating ecdysone-responsive genes [20], while in *Tribolium castaneum*, it directly activates chitinase genes during molting [21]. Similarly, *Locusta migratoria* HR3 controls chitin metabolism during larval–pupal transitions [22], and *Blattella germanica* HR3 governs early embryogenesis via interactions with EcR/USP [23]. The *B. mori* HR3 (BmHR3) shares high homology with *Drosophila* HR3 and is critical for ovary and wing development [24,25,26]. Despite its roles in organogenesis and metamorphosis, whether BmHR3 regulates SG development and fibroin synthesis—a process central to sericulture—remains unknown. This gap prompted us to investigate BmHR3’s function in *B. mori* SGs.

Previous studies indicate that 20E signaling regulates autophagy and apoptosis in SG cells [27,28,29]. We hypothesize that dysregulated *BmHR3* expression impairs SG development and fibroin synthesis. To verify this, we overexpress and deplete the *BmHR3* in the PSG of *B. mori*, respectively. Consequently, dysregulation of *BmHR3* causes severe developmental defects in the PSG, as well as significant repression of silk fibroin gene expression, indicating that BmHR3 functions as a negative regulator in the PSG. This work reveals the crucial role of BmHR3 and provides useful guidance for understanding the mechanism by which 20E signaling regulates insect silk-secreting organs.

## 2. Materials and Methods

### 2.1. Silkworm Strains

The wild-type (WT) silkworm strain *Nistari* was maintained at the State Key Laboratory of Resource Insects (Southwest University, China). Silkworm larvae were reared with fresh mulberry leaves under standard conditions at 25 ± 1 °C, 70–80% relative humidity. The fertilized eggs were collected for DNA injection to generate transgenic silkworms.

### 2.2. Plasmid Construction

The optimized Gal4/UAS binary system [30] was used to generate corresponding transgenic expression vectors. Briefly, the sequences encoding BmHR3 (NCBI accession no. NM_001043547) and the CRISPR/Cas9 sequences (Cas9-coding sequence linked to two sgRNAs) for targeting *BmHR3* were commercially synthesized (GenScript, Nanjing, China) and inserted into the multiple cloning site (MCS) of the pBac [10× UAS-mcs-Ser1pA, 3× P3ECFP] vector to generate the final constructs, named UAS-HR3 and UAS-HR3-sgRNA, respectively (Figure S1A).

### 2.3. Generation of Transgenic Silkworms

The purified DNA of UAS-HR3 and UAS-HR3-sgRNA was microinjected into fertilized silkworm eggs, respectively. Positive broods (UAS transgenes) were subsequently screened with a fluorescence stereomicroscope (Olympus, Tokyo, Japan). To generate Gal4/UAS transgenes, the UAS transgene was crossed with fibH-Gal4 (HG4, a PSG-specific Gal4 line driven by the *fibH* promoter) [30] to produce offspring, and Gal4/UAS transgenes were obtained by screening silkworm eyes by double fluorescence. To detect knockout lines, the PSGs of Gal4/UAS larvae were dissected to extract genomic DNA using a tissue DNA kit (Omega, Irving, TX, USA). Genomic PCR amplification was performed using target-specific primers (Table S1). The resulting PCR products were inserted into the pMD19-T Simple vector (Takara, Osaka, Japan), sequenced (Sangon Biotech, Shanghai, China), and analyzed by comparison with the original sequence of *BmHR3*.

### 2.4. Real-Time Quantitative PCR (RT-qPCR)

The PSGs of silkworm larvae were dissected, and total RNA was extracted using a tissue RNA kit (Omega, Irving, TX, USA). cDNA templates were subsequently synthesized using the PrimeScript RT Reagent Kit and gDNA Eraser (Takara, Osaka, Japan). TB Green Premix Ex Taq II was used to perform qRT-PCR in a 20-μL system, which included 2 μL of cDNA template, 1 μL of SYBR@Green I, 0.4 μL of ROX reference dye (Takara, Osaka, Japan), and 0.8 μL of each primer. A 7500 FAST Real-Time PCR system (ABI, Los Angeles, CA, USA) was used to perform the reactions. The *B. mori* housekeeping gene *BmeIF4A* [31] was used as the internal control. The sequences of the primers that were used in this study are shown in Table S1. All the experiments were carried out with three biological replicates. All the data were analyzed using GraphPad Prism 9.0.

### 2.5. Immunostaining

We prepared newly hatched silkworm larvae (1L1D) in groups of 20 and dissected whole SGs in 1× PBS under a microscope. The samples were placed on a glass slide with a small drop of 1× PBS, fixed with 4% paraformaldehyde for 15 min, and washed 3 times with 1× PBS. The samples were permeabilized with Immunostaining Permeabilization Buffer and Triton X-100 (Beyotime, Haimen, China) for 15 min and then washed 3 times with 1× PBS. The nuclei were stained with DAPI solution (Beyotime, Haimen, China). A small amount of Antifade Mounting Medium was added to the stained SGs, which were then stored in the dark after mounting. Images were captured with an inverted fluorescence microscope (Olympus, Tokyo, Japan) and analyzed.

### 2.6. Dual-Luciferase Reporter Assay

The luciferase reporter plasmid was constructed by inserting the promoter sequence into the PGL4 base vector (Promega, Madison, WI, USA). The plasmid vector carrying the truncated promoter was commercially synthesized (GenScript, Nanjing, China), and a luciferase assay was performed in cultured *B. mori* embryonic cells (BmE) using the promoter luciferase reporter gene described above. Reporter plasmids (2 μg) were transfected with Renilla (0.2 μg) to determine the transfection efficiency. For the luciferase assay with the overexpression plasmid, cells were transfected with the overexpression and reporter plasmid DNA for 36 h and then harvested for subsequent assays. All the data were analyzed using GraphPad Prism 9.0.

### 2.7. Electrophoretic Mobility Shift Assay (EMSA)

Oligonucleotides containing binding sites were commercially synthesized (Sangon Biotech, Shanghai, China), labeled with biotin at their 5′ ends, incubated at 95 °C for 10 min, and then annealed to generate double-stranded probes. Unlabeled and biotin-labeled mutant probes were used as competition probes and visualization probes, respectively. The sequences of the WT and mutant probes are listed in Table S1. After transfection for 48 h, the BmE cells were harvested and washed with 1× PBS. Nuclear proteins were extracted using a nuclear and cytoplasmic protein extraction kit (Beyotime, Haimen, China), and EMSAs were performed using a chemiluminescent EMSA kit (Beyotime, Haimen, China). Subsequent experiments were performed according to the manufacturer’s instructions. The promoter sequence of each target gene was obtained from KAIKObase (http://kaikobase.dna.affrc.go.jp, accessed on 10 October 2018) and verified by sequencing. Potential binding sites for BmHR3 were identified in a study by Kageyama Y et al. [32].

### 2.8. Statistical Analyses

**Figure 1:**
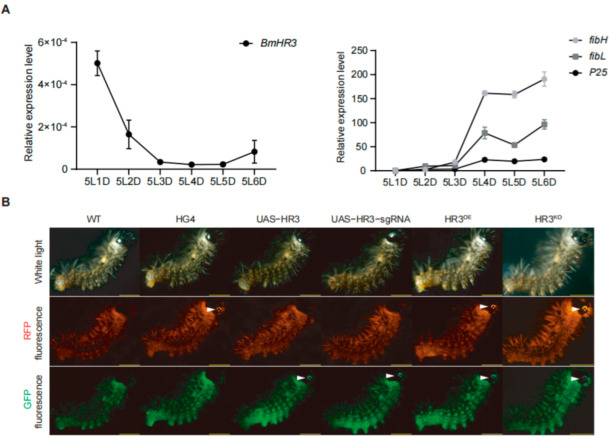
Tissue-specific overexpression and knockout of *BmHR3* in silkworm posterior silk glands (PSGs). (**A**) Temporal expression patterns of *BmHR3* and silk fibroin-coding genes (*fibH*, *fibL*, and *P25*) in wild-type PSGs during the fifth instar. 5L1D~5L6D, day 1 to day 6 of the fifth-instar larvae. (**B**) Fluorescence screening of transgenic silkworms. White arrows indicate eye-specific fluorescence (3× P3-ECFP and 3× P3-RFP markers). Data are shown as mean ± SD (n = 3 biological replicates). Significant differences between the treatment and the control were determined by Student’s *t* test.

**Figure 2:**
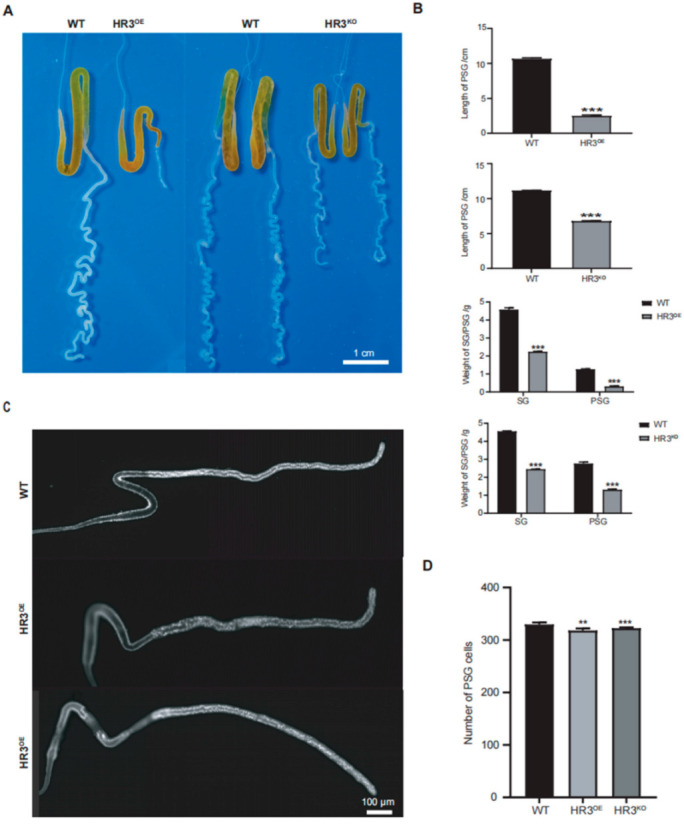
Developmental defects in PSGs of *BmHR3*-dysregulated silkworms. (**A**) Morphological comparison of PSGs from HR3^OE^, HR3^KO^, and WT silkworms at 5L6D. Scale bar: 1 cm. (**B**) Quantification of PSG length and weight (n = 30 silk glands per group). (**C**) DAPI staining of PSG cells in newly hatched larvae (1L1D) of HR3^OE^ and HR3^KO^ silkworms. Scale bar: 100 μm. (**D**) PSG cell number quantification (n = 10 silk glands per group). Data are shown as mean ± SD (n = 3 biological replicates). Significant differences vs. WT were determined by Student’s *t* test (**, *p* < 0.01; ***, *p* < 0.001).

**Figure 3:**
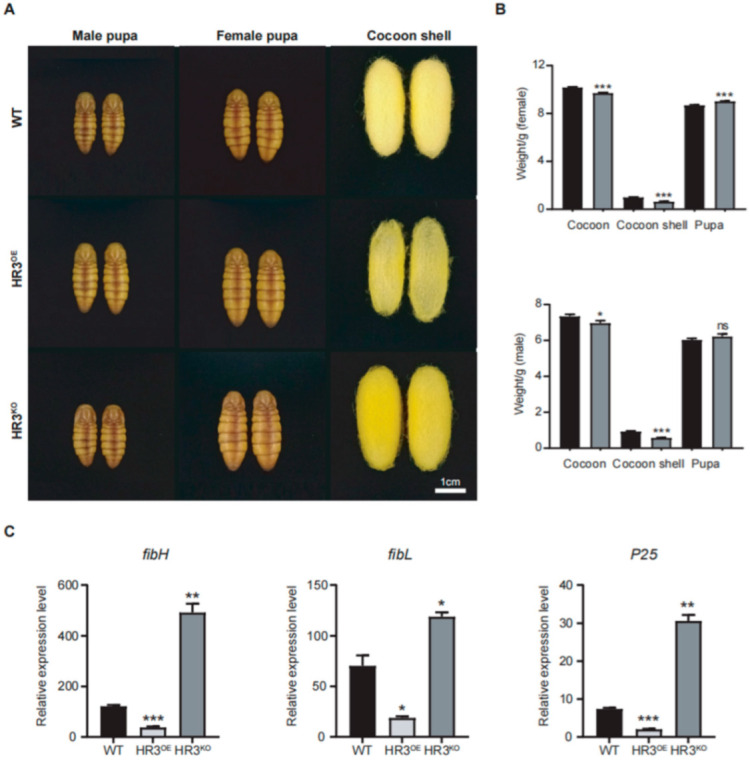
*BmHR3* dysregulation impairs silk fibroin synthesis. (**A**) Phenotypes of pupae and cocoon shells from HR3^OE^, HR3^KO^, and WT silkworms. Scale: 1 cm. (**B**) Cocoon shell and whole cocoon weight quantification of HR3^KO^ and WT silkworms (n = 15 cocoons per group). (**C**) Relative mRNA levels of *fibH*, *fibL*, and *P25* in PSGs. Data are shown as mean ± SD (n = 3 biological replicates). Significant differences vs. WT were determined by Student’s *t* test (*, *p* < 0.05; **, *p* < 0.01; ***, *p* < 0.001, “ns” stands for not significant).

**Figure 4:**
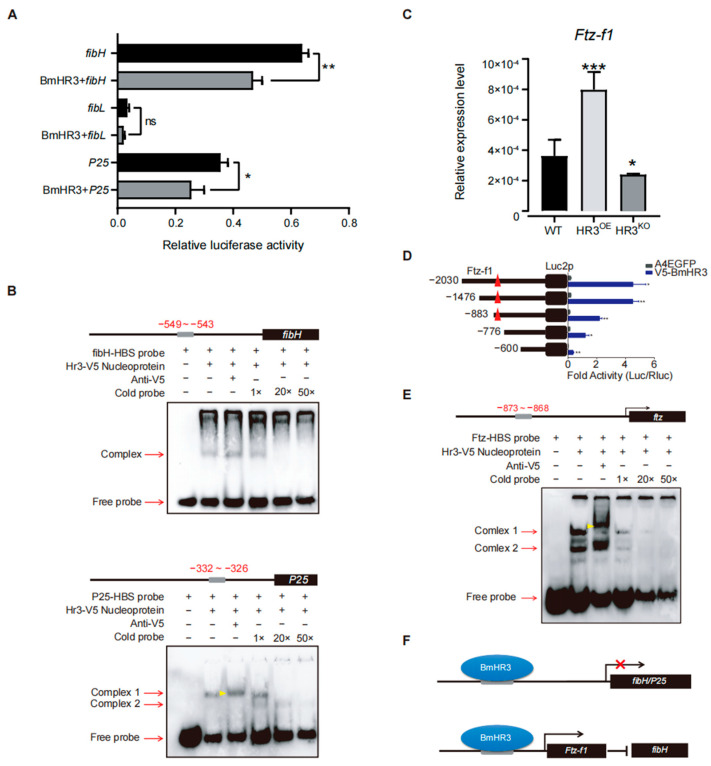
BmHR3 regulates fibroin genes through direct and indirect mechanisms. (**A**) Dual-luciferase reporter assay showing BmHR3-mediated repression of *fibH* and *P25* promoters in BmE cells. Reporter constructs containing the promoters of *fibH*, *fibL*, and *P25* were separately co-transfected with BmHR3-V5. Luciferase reporters containing the promoters of *fibH*, *fibL*, and *P25* were used as controls, respectively. The relative activity was calculated according to the firefly luciferase/Renilla luciferase ratio. (**B**) EMSA confirming direct binding of BmHR3 to *fibH* (−549 to −543 bp) and *P25* (−332 to −326 bp) promoters. Nucleoproteins were extracted from BmE cells that were transfected with BmHR3. The concentrations of the competitors were 100 nM (1×), 2 μM (20×), and 5 μM (50×). The gray squares represent potential binding areas; the black square represents the CDS region; the red arrows represent the binding bands and free probe bands; the yellow arrowhead represents the shift bands. (**C**) *Ftz-f1* mRNA levels in PSGs of HR3^OE^ and HR3^KO^ silkworms. (**D**) Ftz-f1 activates *fibH* promoter activity in BmE cells. Reporter constructs containing the promoters of *fibH* were co-transfected with Ftz-f1-Flag. A luciferase reporter containing the *fibH* promoter was used as a control. The relative activity was calculated according to the firefly luciferase/Renilla luciferase ratio. (**E**) EMSA showing BmHR3 binding to the *Ftz-f1* promoter (−873 to −868 bp). Nucleoproteins were extracted from BmE cells that were transfected with BmHR3. The concentrations of the competitors were 100 nM (1×), 2 μM (20×), and 5 μM (50×). The gray squares represent potential binding areas; the black square represents the CDS region; The red arrows represent the binding bands and free probe bands; the yellow arrowhead represents the shifted bands. (**F**) Model of BmHR3-mediated regulation: Direct inhibition of *fibH* and *P25*, and indirect suppression via *Ftz-f1*. The red symbol × represents the repression of gene transcription. Data are mean ± SD (n = 3). Significant differences vs. control: *, *p* < 0.05; **, *p* < 0.01; ***, *p* < 0.001 (Student’s *t* test).

Statistical analysis was conducted using GraphPad Prism v9.0 (GraphPad Software, San Diego, CA, USA). Experimental procedures included: (1) RT-qPCR quantification of target gene expression in SGs; (2) morphometric measurements (weight and length) of PSGs; (3) PSG cell counting; (4) gravimetric analysis of cocoons (shell, whole cocoon, and pupa); and (5) dual-luciferase reporter assays. All experiments were performed with three biological replicates. Data represent mean ± SD from three independent experimental trials. Between-group comparisons were analyzed by Student’s *t*-test with significance thresholds set at * *p* < 0.05, ** *p* < 0.01, and *** *p* < 0.001 (applied to Figure 1A, Figure 2B,D, Figure 3B,C, Figure 4A,C and Figure S1B). Graphical representations were generated using GraphPad Prism v9.0, unless specifically noted.

## 3. Results

### 3.1. Tissue-Specific Overexpression and Knockout of BmHR3 in Silkworm Silk Glands

We first analyzed the temporal expression of *BmHR3* in the PSG of fifth-instar larvae (Figure 1A). The mRNA level of *BmHR3* peaked on day 1 of the fifth instar (5L1D), then rapidly decreased and remained at a low level until 5L6D. This expression pattern was inversely correlated with the three silk fibroin genes (*fibH*, *fibL*, and *P25*), suggesting that BmHR3 may negatively regulate fibroin protein synthesis during the feeding stage. To verify this, we generated transgenic silkworms harboring UAS-HR3 and UAS-HR3-sgRNA, respectively (Figure 1B). The moths carrying the UAS transgene were separately crossed with those carrying the fibH-Gal4 transgene (HG4), resulting in the generation of Gal4/UAS transgenic silkworms characterized by *BmHR3* overexpression and knockout, which were referred to as HR3^OE^ and HR3^KO^, respectively (Figure 1B). Furthermore, we validated the overexpression and knockout effects of *BmHR3* in transgenic individuals at the transcriptional level. The results showed that *BmHR3* was efficiently overexpressed and knocked out in the PSGs of HR3^OE^ and HR3^KO^ individuals (Figure S1B,C), and these individuals could be used for subsequent experiments.

### 3.2. Dysregulation of BmHR3 Results in Severe Developmental Mutations in SGs

Given the large size and readily observable organ morphology of final-instar larvae, we initially examined SG morphology in HR3^OE^ and HR3^KO^ silkworms at this developmental stage. Notably, the PSGs of both HR3^OE^ and HR3^KO^ silkworms exhibited significantly reduced length and diameter compared to WT controls. HR3^OE^ PSGs displayed straightened morphology lacking characteristic curvature, while HR3^KO^ PSGs showed exaggerated curvature with continuous protrusions forming nodular structures, particularly in the anterior region (Figure 2A). Quantitative measurements revealed a 76.53% reduction in PSG length (*p* < 0.001) and an 86.01% decrease in weight (*p* < 0.001) for HR3^OE^ mutants. Similarly, HR3^KO^ PSGs demonstrated a 39.01% length reduction (*p* < 0.001) and a 46.53% mass loss (*p* < 0.001) relative to WT (Figure 2B). Further analysis of newly hatched larvae showed comparable PSG lengths between mutants and WT (*p* > 0.05), but HR3^OE^ and HR3^KO^ larvae exhibited significantly reduced PSG cell numbers (*p* < 0.01) (Figure 2C,D), indicating BmHR3’s involvement in PSG cell cycle progression. These findings collectively demonstrate that BmHR3 is essential for normal PSG development and that its dysregulation causes severe morphological defects.

### 3.3. Dysregulation of BmHR3 Severely Affects Silk Fibroin Synthesis

Morphometric analysis revealed abnormal cocoon phenotypes in HR3^OE^ and HR3^KO^ silkworms (Figure 3A,B). The cocoon shells of HR3^OE^ silkworms were markedly thinner and structurally fragile. HR3^KO^ cocoons showed a 35.7% and 37.4% reduction in female and male cocoon weights, respectively (*p* < 0.0001) (Figure 3B). Interestingly, pupae from both mutant strains exhibited increased size compared to WT controls, likely resulting from impaired silk protein secretion and trafficking. Furthermore, we quantified the expression levels of silk fibroin genes (*fibH*, *fibL*, and *P25*) in the PSGs of these mutants. HR3^OE^ silkworms displayed significant reductions in *fibH*, *fibL*, and *P25* transcript levels (*p* < 0.05), whereas HR3^KO^ silkworms showed pronounced upregulation of these genes (*p* < 0.05) (Figure 3C). These findings collectively demonstrate that BmHR3 is essential for regulating silk protein synthesis.

### 3.4. BmHR3 Regulates Silk Fibroin Genes via Direct and Indirect Mechanisms

To elucidate the transcriptional regulatory mechanisms of BmHR3, we performed dual-luciferase reporter assays in BmE cells by cotransfecting BmHR3 expression vectors with promoter constructs of *fibH*, *fibL*, and *P25* (Figure 4A). Quantitative analysis revealed that BmHR3 significantly suppressed *fibH* (*p* = 0.0016) and *P25* (*p* = 0.027) promoter activities, whereas *fibL* promoter activity remained unchanged (*p* > 0.05). This transcriptional repression pattern suggested direct regulation of *fibH* and *P25* by BmHR3. Bioinformatic analysis of fibroin promoters identified conserved HR3-binding sites (HBS) in *fibH* (−549 to −543) and *P25* (−332 to −326), designated as fibH-HBS and P25-HBS, respectively. EMSAs confirmed sequence-specific binding of BmHR3 protein to both HBS motifs (Figure 4B). Given the established regulatory relationship between Ftz-f1 and *fibH* [15], we investigated potential interactions between BmHR3 and Ftz-f1. RT-qPCR analysis showed significant upregulation of Ftz-f1 transcripts in HR3^OE^ PSGs (*p* < 0.0003) and downregulation in HR3^KO^ silkworms (*p* < 0.01) (Figure 4C). Subsequent dual-luciferase assays and EMSAs demonstrated direct binding of BmHR3 to a consensus site (−873 to −868) within the Ftz-f1 promoter (Figure 4D,E). Collectively, these results indicate that BmHR3 directly regulates silk fibroin gene expression and indirectly suppresses *fibH* through modulation of *Ftz-f1* expression, as summarized in Figure 4F.

## 4. Discussion

Our study identified BmHR3 as a key 20E-response factor that regulates the specialized SGs of *B. mori*. Through targeted genetic manipulation of PSGs, we demonstrated that BmHR3 regulates both the morphological development of the PSG and the molecular regulation of fibroin protein synthesis—a crucial role that has not previously been characterized in silk-secreting insects.

Severe silk gland dysplasia was observed in both the overexpression and knockout lines (Figure 2), revealing the essential role of BmHR3 in the regulation of SG growth and development. Moreover, dramatic changes in the transcription of silk fibroin genes were observed upon BmHR3 overexpression and knockout (Figure 3C), indicating that BmHR3 is a dominant regulator in PSGs. This finding is consistent with previous findings in other insect organs, namely, that HR3 plays a crucial regulatory role during organ development [20,21,22,23,33]; however, our findings, including evidence that BmHR3 binds to fibroin gene promoters (Figure 4A,B), extend its function beyond its conventional role in regulating molting and metamorphosis.

The core binding motifs of BmHR3 we identified in the *fibH* and *P25* promoters (Figure 4B) share strong homology with the *Drosophila* HR3-binding site (HBS, 5′-GGGTCA-3′) [32]. This conservation suggests that HR3’s regulatory mode on gene transcription may be evolutionarily retained across insect species. While our results support in vitro binding, future in vivo assays would provide stronger evidence of direct transcriptional regulation of BmHR3. In addition, HR3 can function by coordinating with other 20E-response factors, such as Ftz-f1 and HR4 [32,34,35,36]. Our results indicate that BmHR3 and Ftz-f1 cooperate to regulate fibroin gene expression, although both proteins can independently bind to the promoter of *fibH* (Figure 4C–E); these results suggest that the diversification of the mechanism underlying BmHR3 regulation is worthy of further study. Moreover, silk production is influenced by epigenetic modifications, such as DNA methylation and histone acetylation, which regulate tissue-specific gene expression [37,38,39,40]. While this study focused on transcriptional regulation of BmHR3, it would be interesting to explore how BmHR3 interacts with epigenetic modifiers to fine-tune fibroin protein synthesis.

The tissue-specific effects of our genetic manipulations have important biological implications. The maintained systemic development in SG-specific overexpression and knockout mutants (no significant differences were observed in cocooning and pupation rates compared with those of the wild type) differs from that in lethal transgenic mutants reported everywhere, highlighting the unique capacity of silkworms to regulate metamorphic processes in an organ-autonomous manner. This evolutionary adaptation likely supports continuous silk production during the larval—pupal transition—a critical feature for sericulture. From an applied perspective, our study also demonstrates the potential for manipulating the activity of regulators, such as 20E-related factors, to establish various silkworm resources that are expected to be applied in fields other than textiles, such as medicine and food.

Our findings that BmHR3 is directly involved in SG development and silk protein synthesis establish a new paradigm for nuclear receptor function in the regulation of insect economic traits, and these results provide guidance for understanding how hormonal signals are translated into tissue-specific synthetic programs—a mechanism that may extend to other silk-producing insects. Future studies should delve deeper into the molecular mechanisms underlying BmHR3-mediated regulation of target genes in SGs. Additionally, the spatiotemporal coordination between BmHR3 and other co-factors or signaling pathways in the regulation of SG development and silk protein synthesis warrants systematic analysis to optimize genetic manipulation strategies for sericultural applications.

## Data Availability

The original contributions presented in this study are included in the article/Supplementary Material. Further inquiries can be directed to the corresponding authors.

## Supplementary Materials

The following supporting information can be downloaded at: https://www.mdpi.com/article/10.3390/insects16040369/s1, Figure S1: Vector construction and molecular validation of transgenic silkworms; Table S1: Primers, probes, and antibodies used in this study.
