# Supplementary material for: BmHR3 Is Essential for Silk Gland Development and Silk Protein Synthesis in Silkworms (Bombyx mori)
Source: Insects. 2025 Apr 1;16(4):369. doi: 10.3390/insects16040369 (PMC12028065; doi:10.3390/insects16040369)
Supplement: Supplementary file 1 [file insects-16-00369-s001.zip › insects-3512651-supplementary.pdf]

Supplementary figures

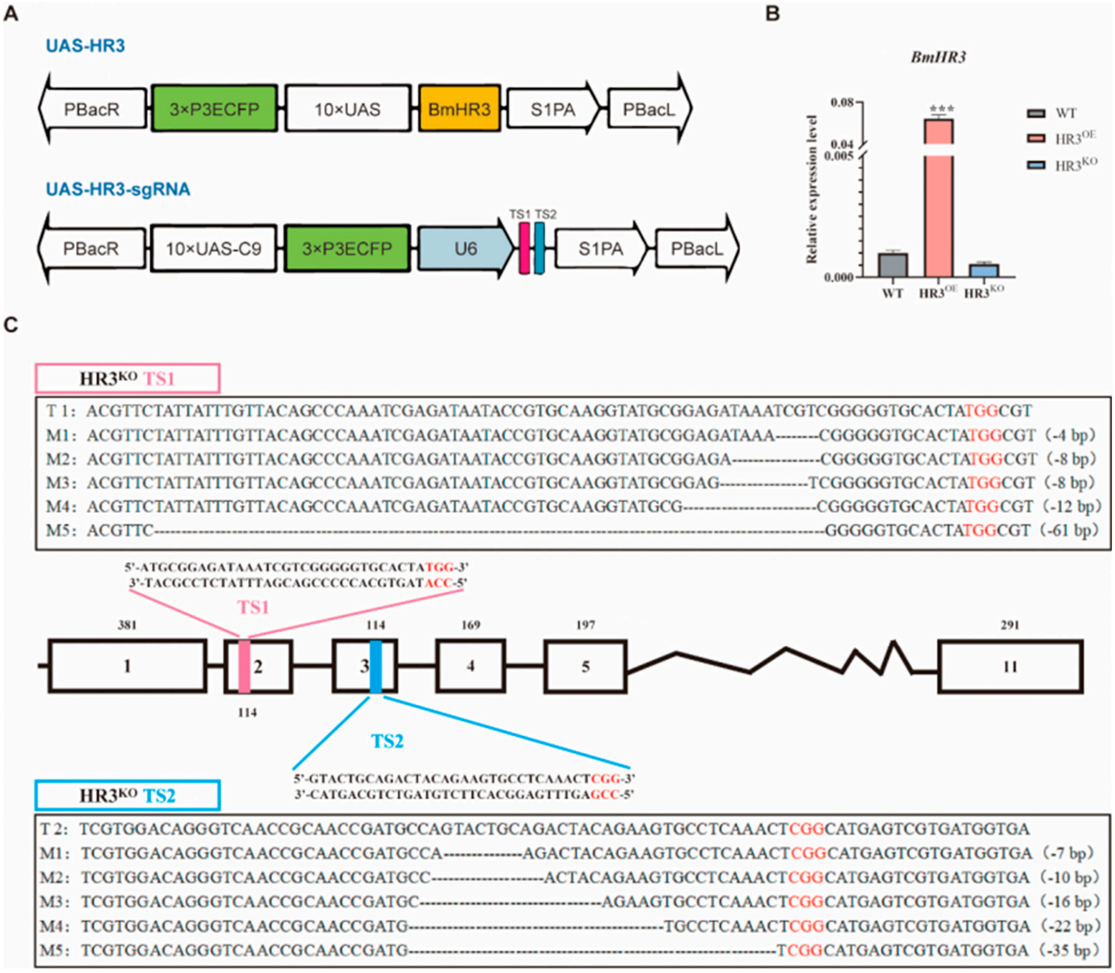

Figure S1. Vector construction and molecular validation of transgenic silkworms. **(A)** Schematic of the constructed expression vectors that were used for *BmHR3* overexpression (UAS-HR3) and knockout (UAS-HR3-sgRNA). 3×P3, eye-specific promoter; ECFP, blue–green (cyan) fluorescence protein marker; yellow square, *BmHR3* coding sequence; pink square, TS1 target; dark blue square, TS2 target. **(B)** Relative mRNA levels of *BmHR3* in the PSGs of $HR3^{OE}$ and $HR3^{KO}$ silkworms. There were three biological replicates (n = 3). Student's t test was used for the data analysis, and asterisks indicate significant differences between the treatment and the control (*, $P < 0.05$; **, $P < 0.01$; ***, $P < 0.001$). **(C)** Sequencing validation of $HR3^{KO}$ knockout mutants. The pink area indicates the base deletion of the TS1 target, and the blue area indicates the base deletion of the TS2 target.

Supplementary Table S1

Name	Sequence (5’-3’)
**Used for genome**	
UAS-genome_F	GTAACCAGCAACCAAGTAAATCA
S1PA-genome_R	GACGCATCAGTGTAATAAATGTGTA
FHp-seqF	ATTCTCCAGGATGGTTGTG
Cas9-genome_F1	AAAAGTAACCAGCAACCAAG
Cas9-genome_R2	CAGTTCGCCAAGATGTATTT
HR3-T1_F	CACACACTTCAGGTGGGAG
HR3-T1_R	GTTGCGAGGACACTGGTAG
HR3-T2_F	AACTACCAGTGTCCTCGCAA
HR3-T2_R	ACCTTCTCTCGCTGCTTCTT
**Used for qPCR**	
Hr3-A-qF	CAGGGTTCTTGGACGCAGACTTC
Hr3-A-qR	AGTCCAACCACATCTCCTCGTAG
fibH-qF	CAGGGGATACGGACAAGGT
fibH-qR	TTCACACAAGGCAGTGCTCT
fibL-qF	GGAGGTGGAAGAATCTATGAC
fibL-qR	TGTAGGCAGCGATGTTGT
P25-qF	GGGTCTGCCCATCTTCCAC
P25-qR	CTCGCCAGCCAGTTCCTCT
Ftz-f1-qF	GCAAAGTGCTGGACCAAAAT
Ftz-f1-qR	GTCAAACTTCTGCCCGTTGT
SW22934-qF	TTCGTACTGGCTCTTCTCGT
SW22934-qR	CAAAGTTGATAGCAATTCCCT
**Used for EMSA**	
fibH-HBS-543	Biotin-TGGTAAGTATTGACCCAAGCTATCACC
anti-FibH-HBS-543	GGTGATAGCTTGGGTCAATACTTACCA
Comp-HBS-543	TGGTAAGTATTGACCCAAGCTATCACC
P25-HBS-326	Biotin-GAAGTCTGCCGGGTCAGCTATTGTACT
anti-P25-HBS-326	AGTACAATAGCTGACCCGGCAGACTTC
Comp-HBS-326	GAAGTCTGCCGGGTCAGCTATTGTACT
Ftz-HBS-873	Biotin-CAAATGAATTGGGTCAGTTGCACGCAT
anti-Ftz-HBS-873	ATGCGTGCAACTGACCCAATTCATTTG
Comp-HBS-873	CAAATGAATTGGGTCAGTTGCACGCAT
**Anti-Body**	**Form, species and Production company**
anti-V5	Rabbit mAb; CST, #13202
